# *In Situ* Atomic-Scale Investigation of Electromigration Behavior in Cu–Cu Joints at High Current Density

**DOI:** 10.1021/acsnano.5c07534

**Published:** 2025-07-31

**Authors:** Hua-Jing Huang, Chien-Hua Wang, Che-Hung Wang, Fang-Chun Shen, Shih-Chi Yang, Jia-Juen Ong, Wei-Lan Chiu, Hsiang-Hung Chang, Chih Chen, Wen-Wei Wu

**Affiliations:** 1 Department of Materials Science and Engineering, 34914 National Yang Ming Chiao Tung University, Hsinchu 30010, Taiwan; 2 Electronic and Optoelectronic System Research Laboratories, 63129 Industrial Technology Research Institute, Hsinchu 30010, Taiwan; 3 Center for the Intelligent Semiconductor Nano-system Technology Research, 34914 National Yang Ming Chiao Tung University, Hsinchu 30010, Taiwan

**Keywords:** high-resolution TEM, in situ HRTEM, electromigration, electroplated copper, 3D IC

## Abstract

Electromigration (EM) poses significant challenges to the reliability of miniaturized devices, particularly three-dimensional integrated circuits (3DICs) operating under high current densities. The EM phenomenon results from atomic-scale mechanisms involving momentum transfer between electron carriers and atoms. In this study, high-resolution transmission electron microscopy (HRTEM) was employed to investigate the atomic-scale behavior of EM in Cu–Cu joints. The analysis revealed that EM initially induced slip along various crystallographic planes, which gradually evolved into a stable slip along specific orientations, dominating the failure. This anisotropic atomic transport led to progressive degradation at the Cu–Cu bonding interface, including void formation and microstructural evolution. This interface depletion has been identified as a critical factor influencing the reliability of 3DIC packaging. Results emphasized the need for optimizing interconnect and interface properties to mitigate EM-induced failures, which is relevant to the development of high-power semiconductor technologies.

## Graphical abstract

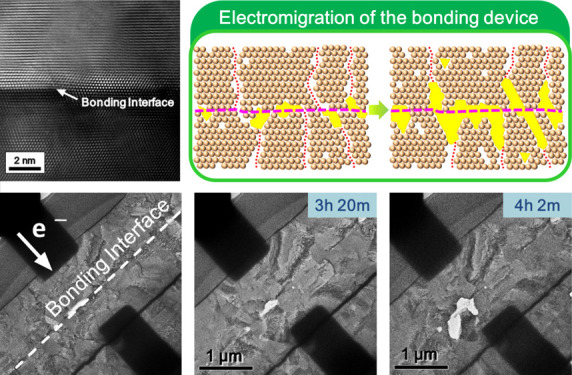

The growing demand for high-performance computing has made integrated circuits (ICs) essential, particularly for applications such as artificial intelligence, data centers, and autonomous driving, that require efficient processing and high-speed data transfer.^1^ These demands have driven the development of advanced packaging technologies, where the need to support higher I/O counts and dense connections creates a high-current-density environment, increasing the risk of electromigration (EM)—when atoms move as they gain momentum from electron carriers.^2^ This significantly affects the durability of fine pitches and poses challenges to future innovations.^3,4^

Copper remains the preferred interconnector material in the semiconductor industry. However, Cu soldering can lead to the formation of intermetallic compounds (IMCs). Although IMCs can enhance the bonding between materials in certain situations, their existence can also result in various issues, including increased contact resistance and long-term reliability concerns.^3–7^ IMCs at the anode reduce circuit efficiency, and delamination caused by material property differences presents a considerable issue. The cathode acts as the source of atoms, and the EM leads to void creation, ultimately resulting in open failure. The drawbacks of this approach stem from the influence of the solder on the overall structure. Previous studies mainly examined the effects of the solder, conductor layer, and IMCs on the joint structure, but such conventional configurations cannot resolve the intrinsic issues of bonding dissimilar materials. Therefore, solder-free bonding is considered a promising approach. Cu-based bonding has been proposed to enable high-performance vertical stacking links with excellent mechanical properties and stable electrical performance. Conventional techniques rely on solder to interconnect wafers, requiring high-temperature processing and subsequent underfill encapsulation to protect the joints and extend service life. In contrast, Cu/SiO_2_ hybrid bonding offers significant advantages by enabling substantial process simplification under appropriate bonding conditions, while achieving precise alignment of the designated redistribution layer (RDL) patterns. This bonding technology involves the direct bonding of wafers with planarized dielectric surfaces and isolated metal interconnections, optimizing bond strength. This technique is pivotal for advancing very large-scale integration (VLSI).^8^ Therefore, advancing high-density interconnects has become imperative, raising concerns about device performance under high-current conditions. EM is triggered during Cu–Cu bonding when subjected to an environment that reaches a threshold current density. Process modifications can optimize the microstructure to improve the EM resistance of the joints. These include altering the parameters, refining the material composition, and adjusting the grain size to promote a more robust interface structure.^9–13^

These treatments can enhance bonding strength and lower energy consumption during manufacturing. However, the bonding interface often cannot be eliminated, causing device failure. Previous studies have explained that the discontinuity between heterogeneous interfaces generates stress fields. This arises from differences in the coefficient of thermal expansion (CTE) and elastic modulus mismatch between materials, which affect the material properties in the interface region.^14,15^ As devices shrink, the challenges of EM at bonding interfaces under high-current density intensify.^3–7,16,17^ As previously mentioned, the development of high-density Cu interconnections is essential, and understanding the mechanisms of EM failure is critical. Nevertheless, studies that directly observe the atomic-level behavior of Cu–Cu joints are limited. In devices, *in situ* transmission electron microscopy (TEM) is recognized as a significant tool. This technique enables not only the inspection of pristine and postreaction states but also the real-time recording of dynamic processes.^18–22^ Continuous imaging yields comprehensive data, thereby aiding in clarifying this phenomenon. As devices continue to scale down, this methodology is particularly relevant for studying fine-pitch interconnects under conditions representative of future high-density integration.

In this study, we aimed to verify the mechanisms of EM failure at the Cu–Cu bonding interface by exploring the *in situ* high-resolution TEM (HRTEM) results in a constant current biasing environment. During the early stages of the current stress, lateral cracking occurred at the bonding interface. As the biasing continued, pronounced void growth was observed. The evolution of these voids, which initially displayed a step-like surface formed by various planes, eventually transformed into a smooth surface dominated by the {111} planes. The substantial void growth observed in specific regions of the sample suggests that this phenomenon, induced by current-dense conditions, should be considered in layout design. This study offers valuable insights into the failure processes of Cu–Cu joints, highlights important perspectives for advanced packaging, and promotes further research into high-reliability devices for the semiconductor industry.

## Results and Discussion

Defined regions on the chip were selected to observe results more intuitively. Each joint was selected independently to ensure no mutual influence (Figure 1a). During Cu electroplating, only the normal vectors of both wafer planes exhibited a high ⟨111⟩ orientation. This characteristic was chosen because Cu atoms diffuse efficiently along the {111} plane owing to their favorable atomic arrangement, with the goal of reducing the thermal budget during fabrication.^23,24^ Under these conditions, periodic reverse currents and specific stirring rates enabled the formation of nanotwinned structures (Figure S1). Previous studies have demonstrated that this type of microstructure offers numerous advantages for addressing line width limitations during device miniaturization,^25^ including high surface diffusivity, low oxidation rate, a resistivity comparable to that of regular Cu, and excellent resistance to EM. The superior EM resistance of such a structure is typically attributed to the triple points, which hinder atomic migration by creating higher energy barriers and altering the diffusion pathways. These features enhance the reliability of the material under high current densities, thereby reducing its degradation.^26,27^ The indeterminate orientation of the horizontal plane of the wafers could result in a noticeable brightness contrast in the S/TEM images owing to the differing diffraction conditions. The scanning transmission electron microscopy (STEM) and energy-dispersive spectroscopy (EDS) image (Figure 1b) shows a visible boundary at a lower magnification. However, based on the elemental distribution of Cu, the interface is continuous and dense, with no apparent voids. A four-point probe measurement was conducted at room temperature and ambient pressure (a schematic of the Kelvin structure is illustrated in Figure S2), which revealed a stable initial resistance, indicating promising bonding quality (Figure 1c). Average initial resistance of each joint was 1.2 mΩ. This low resistance stems from tight bonding, with some areas showing no presence of the bonding interface (Figure 1d,e). This outcome is desirable because a distinct interface can potentially lead to problems during subsequent durability testing.^28,29^

**Figure 1:**
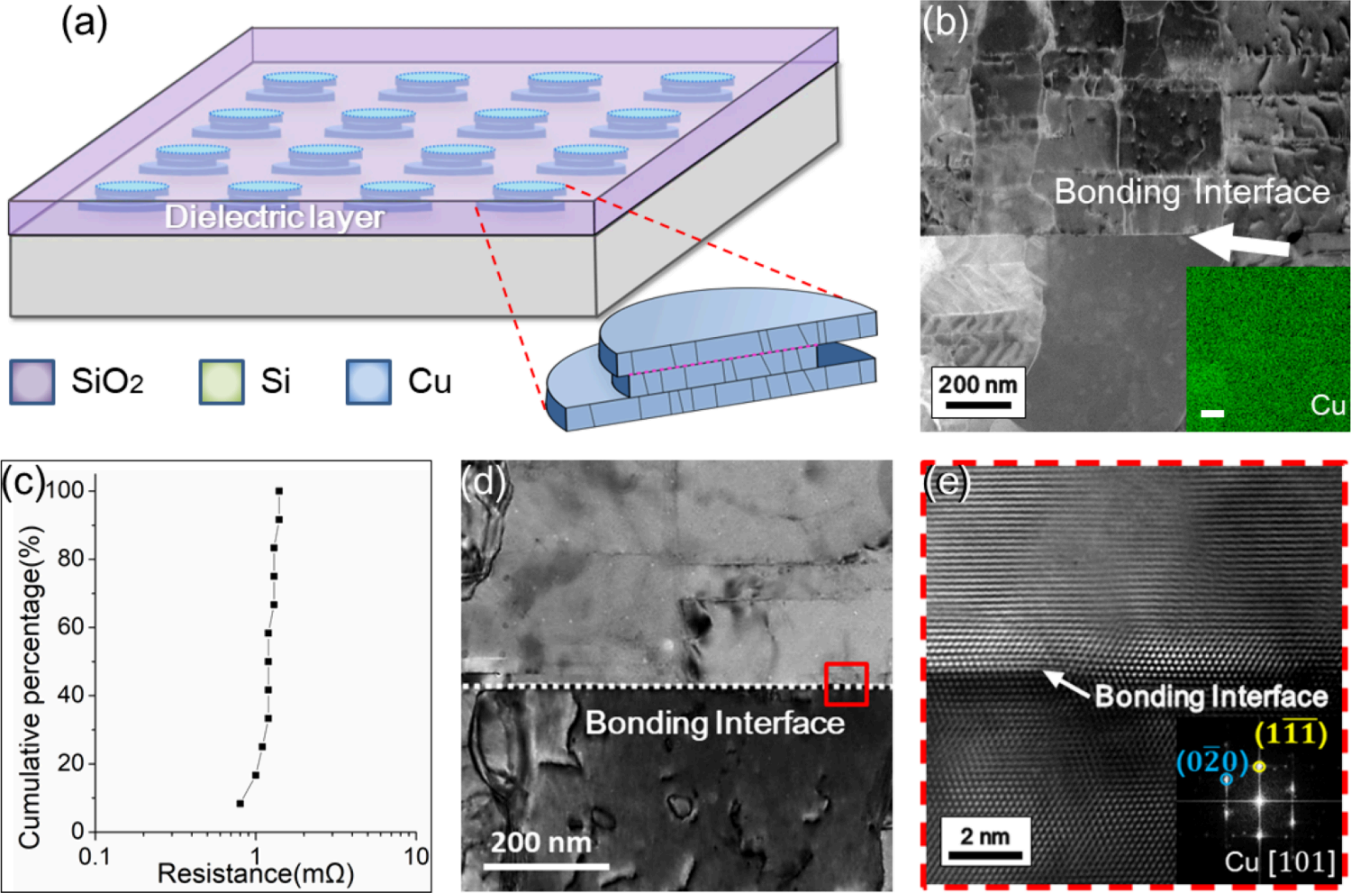
Basic identification of the structure. (a) Schematic of the Cu–Cu bonding. (b) STEM image of the specimen with the Cu-EDS inset. The scale bar in the inset matches that of the STEM image, measuring 200 nm. (c) Average initial resistance on a single Kelvin joint. (d) Cross-sectional TEM image of Cu–Cu bonding. (e) HRTEM image at the location marked in (d). The inset shows the FFT-DP of (e).

Interfaces created by wafer stacking have become a crucial factor in device reliability owing to the advancement of three-dimensional ICs (3D ICs). In the manufacturing process, it is often difficult to eliminate bonding interfaces completely. Compared with perfect crystals, these interfaces are inherently weaker, accumulate defects, and typically possess more free surfaces. Cu atoms tend to migrate along these free surfaces, making atomic movement at the interfaces a significant concern for interconnection reliability.^30,31^ Additionally, the larger surface area and higher proportion of free surfaces of the TEM lamella, compared with traditional wires, result in a lower current density required to trigger EM, as noted in literature.^32,33^ The current density was 10^5^ A/cm^2^, with electrons flowing perpendicular to the bonding interface. Since the TEM sample contained only a Cu–Cu direct bonding region, material mismatch and related interfacial effects are excluded from the present discussion. Under this assumption, the system can be regarded as homogeneous, with the current density assumed to be uniformly distributed across the lamellar specimen. Figure 2a–d shows a series of TEM images depicting voids extending in the direction of the electron wind. During *in situ* current stressing, images were captured at intervals, revealing a linear correlation between the void area and time (Figure 2e). Based on earlier studies, this failure behavior is largely attributed to creep. Theoretically, materials subjected to a constant load or stress over time undergo a gradual deformation process, known as creep, that involves atomic diffusion under stress.^18^ In EM, atomic migration occurs due to momentum transfer from the electron flow driven by electrical stress, leading to a similar diffusion behavior. Creep results in material degradation over time as atoms migrate away from the stressed region, ultimately forming voids or cracks. During EM, atoms are displaced along the electron path, resulting in the formation of voids and hillocks within the material.

**Figure 2:**
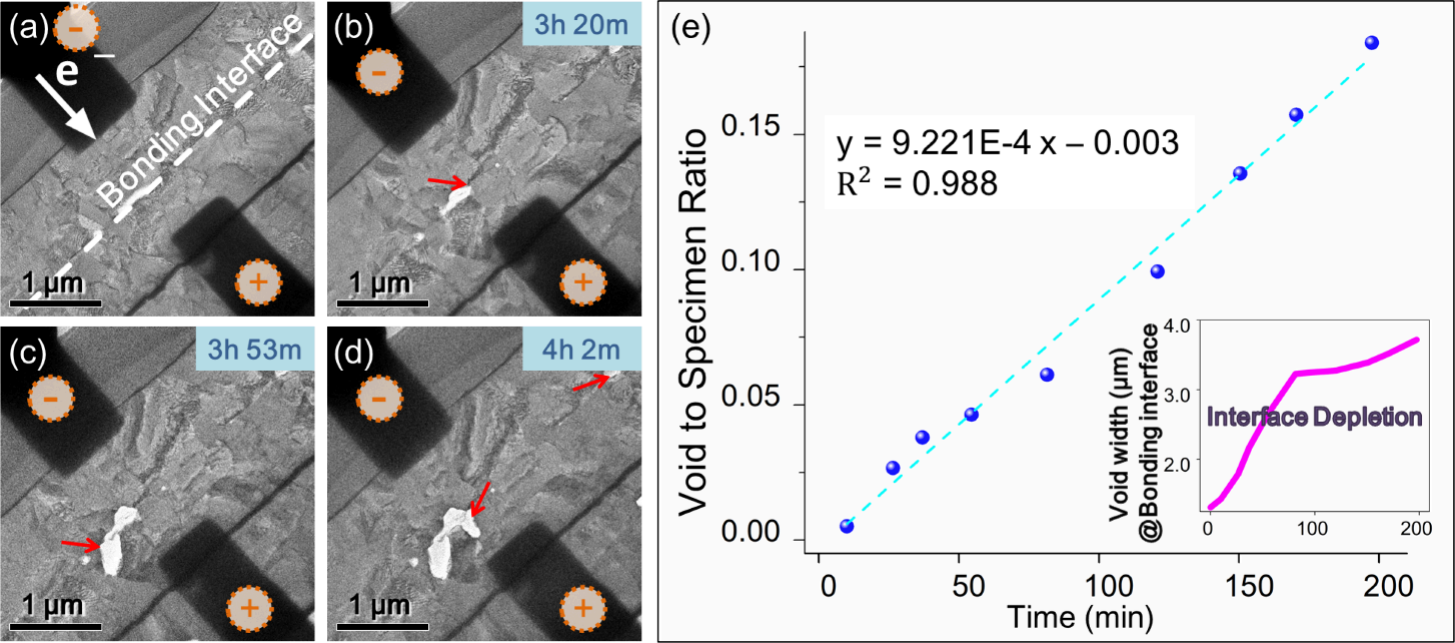
Impact of electron wind on interconnect failure. (a–d) TEM images showing void expansion during *in situ* electrical experiment. White arrow presents the direction of electron flow. (e) Plot of the void-to-specimen area ratio versus stress time with a current density of 10^5^ A/cm^2^. The inset illustrates interface depletion ratio during current-stressing time.

As observed in Figure S3, Cu hillocks appear at the anode. A TEM lamella, in free-standing contact with the membrane of the chip, allows Cu atoms to move unrestricted around the sample as they are carried away from their original positions by the electron wind, resulting in void formation. A portion of the Cu is present at the electrodes in the form of a Cu–Pt alloy, while a greater amount appears as elemental Cu at the anode. These effects compromise the structural integrity of the interconnects, leading to failures such as open or short circuits.^29,34^ Figure 2e illustrates the dependency between the wireless area and time, with the inset showing the ratio of void-induced damage to bonding interface. Initially, damage to the interface occurred rapidly under the applied current stress; however, depletion slowed in the later stages of the experiment. We theorize that horizontal degradation occurred first at the bonding interface. Once the interface degradation reached a certain threshold, the driving force is exacerbated, causing more gradual void growth along the direction of electron flow in the next stages of failure. To clarify whether these results were influenced by the electron beam induction, additional TEM lamellar samples with the same specifications were prepared. Resistance measurements were conducted at room temperature under a prolonged constant voltage of 7.5 V, achieving the same current density (10^5^ A/cm^2^), to enhance data reliability (Figure S4). Retention performance of the samples aligned with those tested under *in situ* conditions, with a similar breakpoint occurring approximately 4 h after the application of the electron current. The initial rapid increase in resistance could be attributed to depletion at the bonding interface, after which resistance stabilized. Based on the resistance growth rate of 7%, calculations using the temperature-resistance equation indicated an approximate 20 °C temperature rise, significantly lower than the conditions required for thermal migration.^35^ To assess the potential influence of even mild heating, we performed separate thermal experiments (Figure S5). During extended *in situ* heating at 50 °C, no notable structural or compositional changes were observed. Figure S5b,d shows indications of slight atomic rearrangement at the bonding interface, suggesting that heating under *in situ* conditions likely facilitates the relaxation or elimination of crystal facets. These findings imply that under ambient conditions, where heat dissipates more readily, any thermal effect would be even less pronounced. Notably, even in the absence of electron beam interference, such working conditions can still trigger EM. In contrast, thermomigration was not detected, possibly due to the limited temperature gradient.

EM-induced failures are inferred to cause severe depletion at the bonding interface. Building on the aforementioned advantages, nanotwinned Cu (nt-Cu) can reduce energy consumption during bonding processes while maintaining high electrical conductivity and enhancing device reliability, both mechanically and electrically. Replacing regular Cu with nt-Cu is an effective approach to improving the EM resistance of Cu-based joints. In this study, devices were designed based on this concept, and the favorable performance of such materials was well demonstrated.^20^ From the smooth regions of the curve in Figure S4, it can be inferred that electro annealing may have occurred during the later stages of biasing.^36–38^ Under these experimental conditions, the lamellar sample reached the threshold required to drive EM. However, the localized increase in current density within the material likely induced local heating, facilitating atomic rearrangement and altering the microstructure. Owing to the intrinsic properties of the material, the observed changes in the crystal orientation or detwinning behavior shown in Figure 2 may be attributed to this phenomenon, potentially extending the lifetime of the interconnects.^39,40^ Theoretically, as the sample dimensions decrease, the failure behavior variability increases, owing to the random distribution of material defects. This variability is further compounded by microstructural sensitivity at the bonding interface, where even subtle changes can alter morphology and degrade device performance.^41,42^ To converge the research themes focusing on atomic migration at the bonding interface, nt-Cu was treated as polycrystalline Cu for analysis. As shown in Figure 2e, during the initial stage of biasing, pronounced void growth degrades the interface. The EM effect caused this notable phenomenon (Figure 3 and Figure S6). The evolution of the reference star positions from the TEM images revealed that two nearby voids exhibited both longitudinal (along the driving force) and transverse (perpendicular to the electron flow) expansions, ultimately merging and resulting in an open circuit.

**Figure 3:**
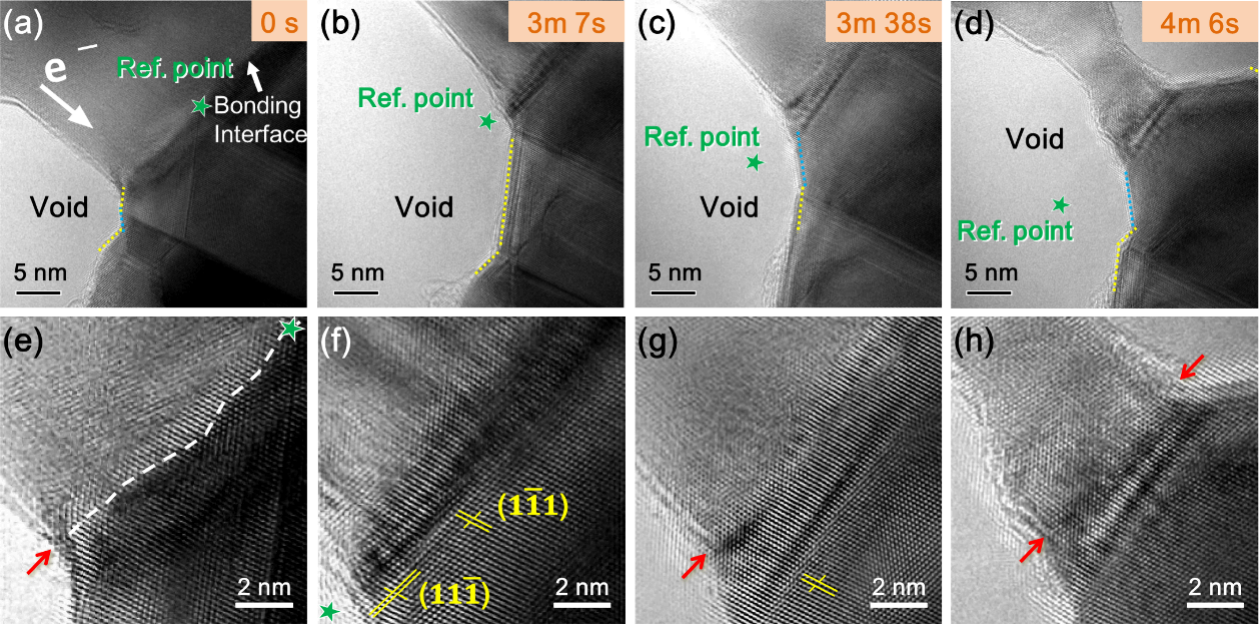
Expansion of the voids along the bonding interface. (a–d) Series of TEM images showing the failure of the bonding interface. White arrow indicates the direction of electron flow. Green stars serve as reference points. (e–h) Atomic-scale TEM images of the bonding interface, enlarged from (a–d). White dotted line indicates the interface. Yellow solid lines denote the atomic arrangement of {111}.

Atomic-scale imaging of the bonding interface shown in Figure 3e–h indicates that the connections at the interface initially appear tightly interwoven with slight waviness, making it difficult to clearly delineate the boundary between the upper and bottom wafers. Previous research explored an athermal method, applying current to induce an electron wind force at the defects, which promotes atomic diffusion, lowers the migration energy barrier, and facilitates atomic rearrangement at lower temperatures.^43^ We suggest that current stress induces atomic migration at the bonding interface, resulting in a more ordered arrangement. The regions indicated by the arrows are inferred to be the interface based on the brightness contrast of the crystal orientations, suggesting good bonding quality. However, the interface is the primary path of degradation owing to its discontinuity. In practical applications, the bonding quality can be determined using electrical measurements. The specific contact resistance (resistance multiplied by the cross-sectional area) for the devices used in this study is approximately 10^–9^ Ω·cm^2^ (Figure 1c), pointing out extremely high electrical efficiency suitable for high-speed electronic devices and high-precision microelectronic applications.

As current stress is applied, degradation occurs along the bonding interface. First, the atomic movement caused by various factors leads to a reduction in the conductive area of the interconnects, which increases the local current density. This significant increase in current density enhances the driving force for EM (*F*_em_). The strain in the grains is believed to be induced by *F*_em_, as defined by the following equation:^44^

$$F_{em}={eZ}^{*}E={eZ}^{*}\rho I$$

where *e* is the electron charge, *Z* ^*^ is the effective charge, *E* is the electrical field, ρ is the resistivity, and *I* is the current density. Additionally, the bonding between the upper and lower wafers creates a discontinuous structure compared to an ideal crystal, which may include various material defects, even when the bonding appears satisfactory. When bonding quality is further compromised due to packaging-related factors, it is possible to infer that voids could exist along the bonding interface. The slightly disordered alignment at the interface may indicate a higher potential energy, making it easier for lattice energy barriers to overcome.^45–47^ Thermodynamically, inadequate bonding significantly enhances overall diffusion behavior. Over time, as the Cu atoms leave, they gradually become thin and develop short-range-ordered {111} arrangements. The {111} planes are prone to slipping and are characterized by the highest atomic packing factor (APF) in face-centered cubic (FCC) structures. Therefore, the bonding strength decreases. Horizontal degradation occurs as atoms continuously exchange momentum with electrons. When approaching an open circuit, atomic imaging allows a clear distinction between the two wafers, revealing a bonding interface resembling that of a grain boundary (GB). During EM, electrons tend to migrate along the GB primarily because these regions provide higher energy-scattering probabilities, enabling more efficient mobility.^33,48,49^ Partial disappearance of the bonding interface was observed in this study. However, the interface still lacked long-range atomic order compared to an ideal crystal, potentially leading to structural discontinuities within the RDL. The right-side void expanded leftward and downward via a {111} slip, ultimately intersecting the left side, representing a scenario that could occur in actual industrial processes. Because the crystal structure is 3D, different misalignments may occur between the microbumps during fabrication, leading to the accumulation of defects at the bonding interface such as vacancies, small-angle GBs, and stacking faults. These factors interact with the dislocation slip, making failure along the bonding interface foreseeable. We speculate that, after a critical point of interface depletion, the longitudinal growth of voids by EM may intensify. Black proposed a model to predict the damage caused by EM in conductive materials, known as Black’s equation:^50^

$$\frac{1}{\mathrm{MTF}}=AJ^{2}\exp\;-\frac{\varphi }{kT}$$

where MTF means median time to failure in hours, *A* is a constant that contains a factor involving the cross-sectional area of the film, *J* is the current density in amperes per square centimeter, φ is activation energy in electron volts, *k* is Boltzmann’s constant, and *T* is temperature in Kelvin degrees. The activation energy was taken from previous literature,^25,51^ and the resulting curve suggests possible failure trends at different temperatures (Figure S7a,b). Calculation details are provided in the Supporting Information.

Black’s equation is typically used to estimate EM lifetimes at the macroscopic scale under various long-term operating conditions, defined by the time to reach steady-state failure. In this study, a single operating condition was used, and experimental parameters along with *ex-situ* data were applied to the equation to assess failure behavior at the atomic scale. The slope in Figure S7a reflects bonding quality at the real-world scale. As previously discussed, inadequate bonding may require considering surface diffusion as a dominant transport mechanism, resulting in a flatter slope and earlier failure at lower temperatures. As a result, the degradation of the bonding interface shortens the failure time of the Cu interconnects.

As this study investigates atomic-scale dynamics in the transient regime of the failure process, the application of Korhonen’s model could offer a more appropriate description. The semi-infinite line solution is as follows:^52^

$$\sigma \left(t\right)=2G\sqrt{\frac{Kt}{\pi }}$$

where σ­(*t*) is the hydrostatic stress as a function of time *t*, *G* is the EM-induced pressure gradient defined as $G=\frac{q^{*}E}{\Omega }$ , *K* is a diffusivity-related constant defined as $K=\frac{D_{a}B\Omega }{kT}$ , and π is the mathematical constant. All parameters were adopted from the literature,^53^ with minor simplifications applied during the calculation. Full details are provided in the Supporting Information.

The time-dependent stress evolution derived from this formula is shown in Figure S7c, revealing a rapid increase in stress over a short period. This results from the model’s assumptions of purely elastic response and infinite line length, which neglect relaxation mechanisms such as plastic deformation, void growth, and GB sliding. In addition, dimensional differences also influence stress development. In practical structures, constraints are often insufficient to sustain the magnitude of back stress predicted by the model. As shown in Figure S7d, the average failure times under two operating conditions are comparable, but the *in situ* tests exhibit a larger standard deviation due to multiple contributing factors. While prior studies have limited applicability at the microscopic scale,^54–56^ they nonetheless serve as a foundation for developing more tailored modeling approaches. In our case, these models were used to explore the speculation that void growth may accelerate after interface depletion. However, the assumptions and scale limitations restrict their relevance at the atomic level.

To gain an in-depth understanding of EM at the bonding interface, atomic-scale imaging was used to reveal the potential mechanisms associated with dislocations (Figure 4 and Figure S8). Initially, the (111), (200), and (311) planes appeared at the free-surface edge. As the Cu atoms migrated, a step-like structure formed, with the (111) plane becoming the primary plane for atomic removal. The free surface continued to evolve along this direction (Figure 4d–f). As shown in Figure 4a–c, the atomic arrangement in the bonding region transitions from slightly disordered to ordered, as the atoms are continuously displaced by the electron flow, causing the interface to gradually thin. Regarding the morphological changes at the free surface, the proportions of various planes at different times were statistically analyzed (Figure S9). Figure S9 shows that the failure is predominantly contributed to by the {111} planes, which is attributed to their status as the closest-packed planes. The {200} and {311} planes demonstrate reciprocal variations, which is consistent with previous studies.^20^ These results suggest that the {311} planes are more significantly affected by the electron flow because they have a larger inner product with a driving force, leading to pronounced fluctuations in their proportion. Conversely, if the {200} planes have a smaller angle with the electron flow, then they exhibit similar notable changes in relative abundance.^57^

**Figure 4:**
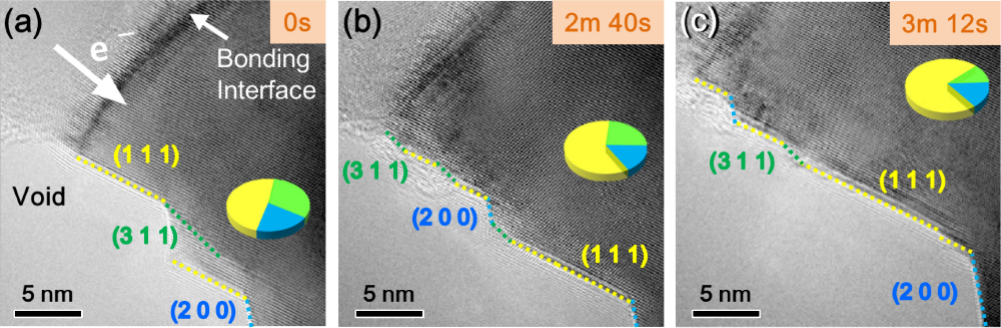
EM phenomenon at the bonding interface. (a–c) Series of TEM images showing the dynamic evolution of the interface in EM. The white arrow indicates the direction of electron flow. Other colors denote slip planes in various directions. Each inset in (a–c) shows the different slip plane proportions for each image.

EM at the bonding interface of the device was clearly demonstrated. To clarify the impact of the bonding interface, we conducted additional in situ experiments using a single upper wafer (Figure 5). Under identical operating conditions, the void growth in the upper wafer exhibited pronounced vertical elongation owing to the EM. As shown in Figure 5a–f, voids grow from the cathode to the anode owing to the electron wind force. The microstructural characteristics led to void extension along the GBs of the columnar grains, evolving from the initial triangular shapes into conical structures. In this experiment, the EDS analysis of the cathode suggested that void formation may be associated with Cu atom loss (Figure 5g–i). The failure behavior differed between experiments. In the single-wafer experiment, considering a continuous crystalline structure without a bonding interface, the electron wind force drove the Cu atom migration, resulting in pronounced vertical void growth. In a complete device, the bonding interface altered void evolution. During the initial stage of current stress, horizontal void growth led to interface depletion. Vertical EM remained significant, further demonstrating the critical role of the bonding interface in 3D ICs. Figure 5j confirms that this phenomenon is primarily induced by the current stress. The regression analysis of void growth over time was compared with that of previous studies.^20^ Compared with the joint with pure Cu, the presence of a bonding interface promoted defect accumulation, leading to earlier failure under similar current densities. The single-wafer experiment exhibited a faster failure rate than expected, likely owing to the increased variability at the reduced observation scale. This study focused on the failure behavior of Cu RDL at the microscopic scale. In contrast, investigations at the macroscopic industrial scale often emphasize the reliability of the entire layout, where failure results from multiple factors. These include constraints imposed by the surrounding dielectric layers, as well as degradation phenomena such as warpage caused by mismatches in the CTE between dissimilar materials, along with other contributing mechanisms. Additionally, the shorter current path may have intensified the Joule heating effect. Given the ongoing miniaturization of devices and rapid reduction in contact spacing and line width, this phenomenon warrants further investigation, particularly to better understand the coupled effects of electrical and thermal fields on bonding interfaces. Moreover, extending such studies to more complex systems could provide valuable insights into practical device applications.

**Figure 5:**
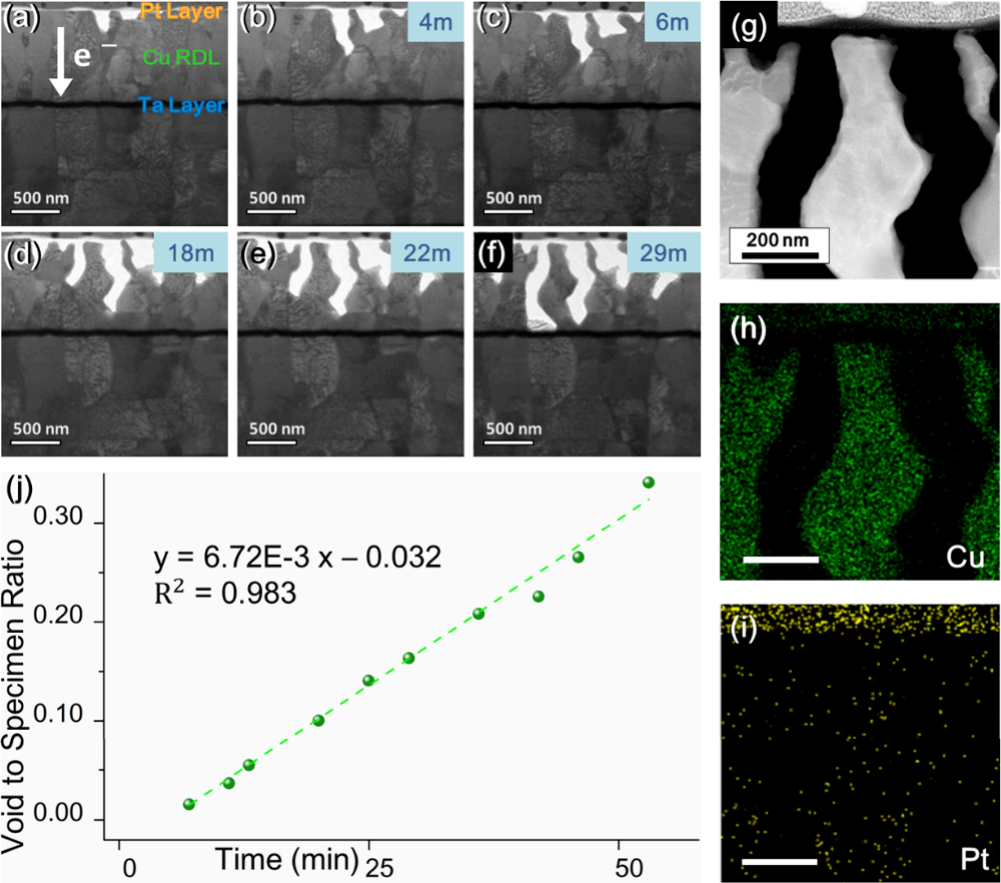
EM phenomenon in the single wafer. (a–f) TEM images showing void expansion in the upper wafer with a current density of 10^5^ A/cm^2^. White arrow presents the direction of electron flow. (g) STEM image of the specimen. (h, i) EDS images of Cu and Pt, respectively. (j) Plot of the void-to-specimen area ratio versus stress time.

The influence of the EM on the joint was observed, including the lateral and vertical void growth. Notably, in the central observation area of the sample, void growth under electrical stress was greater than that in other regions, suggesting a maximum current stress (Figure 6a,b). As previously mentioned, the linear dependence of the area growth on time confirms that it is an EM-induced failure. Variations in void growth across different regions were documented, likely due to initial conditions such as void and grain boundary distributions (Figure S10). The initial state of the device consists of a staggered, dense bonding interface; however, prolonged current stress induces an ordering transition owing to atomic migration, altering the mechanical properties and causing depletion at the interface. This reduces the cross-sectional area of the interconnects, thereby intensifying EM (Figure 6c). The single wafer exhibited well-known EM behavior, causing it to fail earlier than expected owing to current heating (Figure 6d). In 3D IC packaging, defect formation, such as voids and grain boundaries at bonding interfaces, inevitably affects device endurance. However, unique processing behaviors were observed. After applying current stress for 3.5 h, the resistance curve leveled off, which drew our attention. Previous studies indicated that strain gradients increase material hardening, thereby reducing slip around voids.^58,59^ The increased strain gradient made EM-induced Cu atom migration increasingly difficult, slowing the increase in resistance. As previously mentioned, atoms in the thicker regions of the sample have a higher migration probability, suggesting a dynamic equilibrium between atom removal and rearrangement, which may represent a relaxation process to minimize energy.^60^ These behaviors potentially enhance the retention performance of the Cu–Cu joints, improving the electron conduction efficiency and reducing energy consumption. Voids formed during processing may expand because of the momentum exchange between electrons and atoms, both laterally and vertically. Lateral (along the bonding interface) failure arises from the inherent electron wind force and defect accumulation at the interface, which accentuates wafer separation over time. This causes an increased local current density, thus affecting the overall joint reliability. As semiconductor devices scale down, the interfaces created by advanced 3D IC packaging processes merit further in-depth studies.

**Figure 6:**
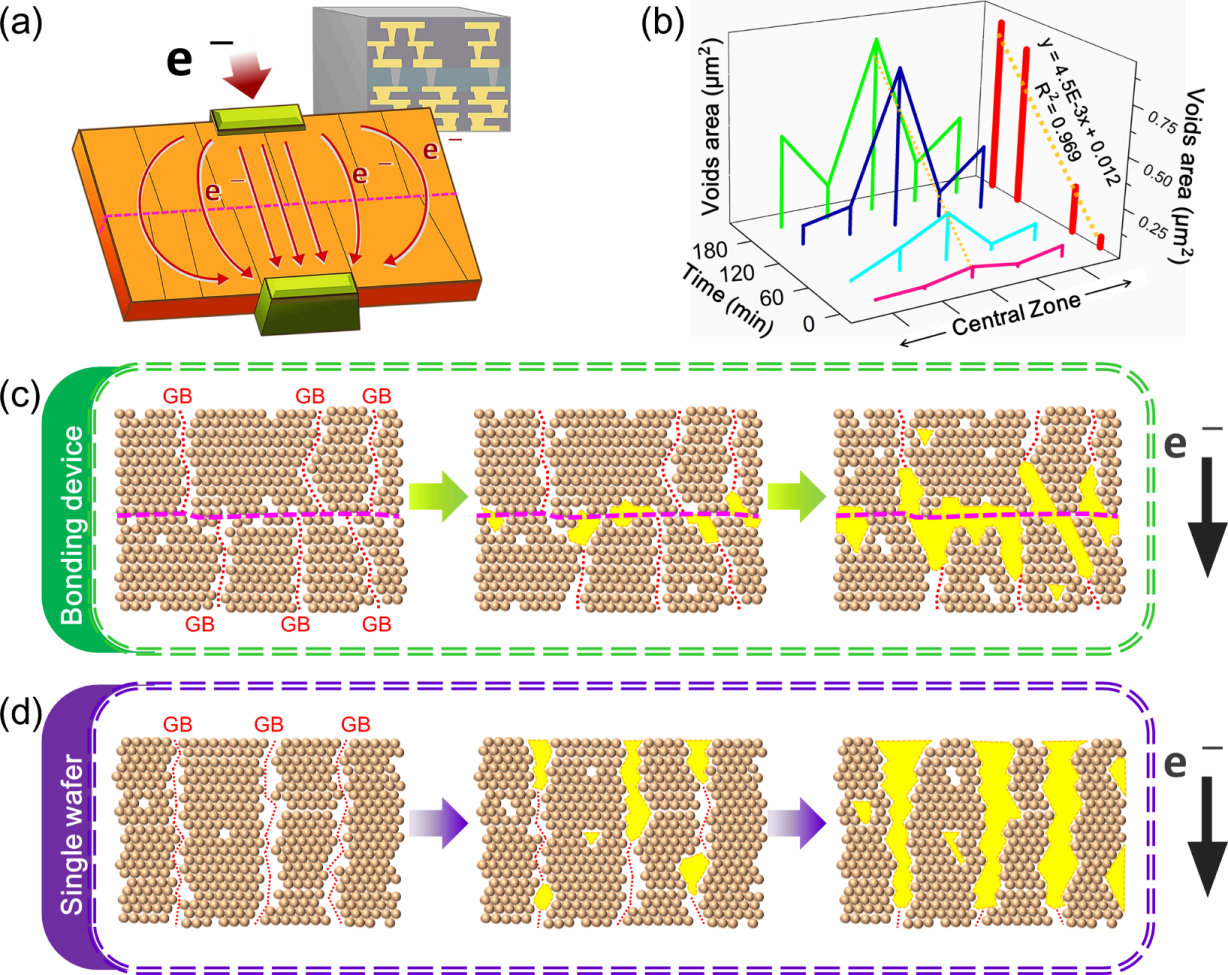
Schematic of the failure mechanism. (a) Representation of EM driving force. (b) Variation of void area changes across different regions. (c, d) Failure mechanisms of bonding device and single wafer, respectively.

## Conclusions

We investigated the significant degradation of Cu–Cu joints under prolonged high current densities. Rapid depletion occurs at the bonding interface in the early stages of current stressing owing to the electron flow altering the atomic arrangements near the interface, which weakens its strength and accelerates collapse. Void growth within Cu further reduces the conductive area, intensifying the EM effects and ultimately leading to open failure. The available data supports the credibility of the experimental results. The discrepancies between microscopic-scale studies and existing EM models point to promising directions for future research. Additionally, Cu atom migration within the device forms hillocks at the anode, which can be observed at the atomic level via *in situ* TEM. At the packaging scale, EM-induced problems can promote diffusion into the dielectric layer, causing material deterioration and short circuits. An obvious absence of conductive pathways appeared in areas directly exposed to DC stress, where the driving force was the highest. Given the ongoing miniaturization of devices, the approach adopted here reflects industrial relevance, as it captures EM behavior under conditions that closely resemble those in future high-density interconnects. This study aims to provide further insights into EM behaviors in 3D IC, contributing to improved reliability in advanced packaging applications, and supports more comprehensive and in-depth investigations in future studies.

## Methods

### Cu/SiO_2_ Hybrid Bonding

First, a silicon dioxide layer was deposited on a 12 in. silicon wafer to serve as the dielectric layer. The designed wire patterns were etched in the regions intended for Cu electroplating, followed by deposition of a Ta/Cu seed layer to form the top wafer, which featured circular Cu vias with a radius of 10 μm. The bottom wafer was fabricated using the damascene process, including additional iterations, to establish a dual-layer Cu structure, consisting of an 8 μm-diameter upper layer stacked above a 12 μm-diameter lower layer. Excess material was removed through chemical–mechanical planarization (CMP) to achieve a planar surface, optimizing conditions for the subsequent Cu/SiO2 hybrid bonding. After CMP, the depth of each Cu layer was approximately 1.25 μm (Figure S11a). The electron backscatter diffraction (EBSD) images of the top wafer revealed a strong ⟨111⟩ orientation (Figure S11b). Because the {111} plane exhibited the highest atomic diffusion rate, this characteristic has the potential to reduce processing costs by lowering the required bonding temperature and vacuum pressure. The bonding surfaces were cleaned using nitrogen plasma and subjected to prebonding under vacuum at a force of 75 kN. Thermal compression bonding occurred at 200 °C, followed by 4 h postannealing at the same temperature to enhance the strength of the Cu–Cu bonding. The high-resolution STEM images indicated that the bonding was dense and nearly continuous, making it challenging to identify the presence of an interface (Figure S11c). Finally, the wafers were diced into small pieces, and each chip contained thousands of joints formed by three Cu layers. Additional circuit configurations, derived from the designed layout and intended for electrical and reliability tests, were implemented for subsequent measurements.

### *In Situ* TEM Observation

The TEM lamellae were prepared using a high-resolution dual-beam focused ion beam (FIB) system (FEI Versa 3D and ZEISS Crossbeam 350) equipped with a scanning electron microscope (SEM). SEM helps control the TEM sample thickness to approximately 90 nm, ensuring the acquisition of high-resolution atomic images. First, a trench was created to confirm the bonding conditions at the observation site. Platinum (Pt) was deposited at the target location to protect the sample from damage by the Ga ion beam, followed by thinning to complete the preparation of the TEM sample. Figure S12 provides additional information about the cross section of the Cu microbump. The STEM electron energy loss spectroscopy (EELS) images indicate that the bonding interface was uniformly distributed and exhibited low oxidation levels, providing adequate support for subsequent experiments. The TEM lamella was transferred to an *in situ* electrical chip using a glass tip, with Pt deposited within the FIB system to serve as an interconnect between the chip and the lamella (shown in Figure S13). Finally, the entire chip was placed in an *in situ* TEM holder (Protochips Audro300). *In situ* TEM videos and images were recorded using a JEOL JEM-F200 system configured for energy-dispersive spectroscopy (EDS) and a OneView CCD camera.

### Measurement of Resistance

Cumulative resistance was measured using a four-point probe on a Keithley 2700 system. The resistance of the TEM lamellar samples was measured using an Agilent 4145 B system by applying a constant voltage of 7.5 V to the specimens for 8 h.

## Supplementary Material



## Notes

Details of the fabrication of Cu–Cu bonding devices and experimental setup; HRTEM images of the bonding interface; and statistical evaluation and other analyses of the results (PDF)
