# Supplementary material for: In Situ Atomic-Scale Investigation of Electromigration Behavior in Cu–Cu Joints at High Current Density
Source: ACS Nano. 2025 Jul 31;19(33):30211–20. doi: 10.1021/acsnano.5c07534 (PMC12392731; doi:10.1021/acsnano.5c07534)
Supplement: Supplementary file 1 [file nn5c07534_si_001.pdf]

Supporting Information

In-situ Atomic-Scale Investigation of Electromigration Behavior in Cu-Cu Joints at High Current Density

*Hua-Jing Huang[1], Chien-Hua Wang[1], Che-Hung Wang[1], Fang-Chun Shen[1], Shih-Chi Yang[1], Jia-Juen Ong[1], Wei-Lan Chiu[2], Hsiang-Hung Chang[2], Chih Chen[1], Wen-Wei Wu[1, 3, *]*

[1]Department of Materials Science and Engineering, National Yang Ming Chiao Tung University, Hsinchu 30010, Taiwan

[2]Electronic and Optoelectronic System Research Laboratories, Industrial Technology Research Institute, Hsinchu 30010, Taiwan

[3]Center for the Intelligent Semiconductor Nano-system Technology Research, National Yang Ming Chiao Tung University, Hsinchu 30010, Taiwan

[*]Correspondence and requests for materials should be addressed to W.-W.W (Email: wwwu@nycu.edu.tw)

List of contents

Figure S1 | Average grain size of the Cu-Cu joint.
Figure S2 | Kelvin structure design layout.
Figure S3 | STEM and EDS analysis images of hillocks.
Figure S4 | *Ex-situ* electrical measurement of TEM sample.
Figure S5 | Characterization after prolonged holding at mild temperature.
Figure S6 | Atomic-scale imaging of different grains.
Figure S7 | Model assessment and data reliability.
Figure S8 | Crystal orientation in observation area.
Figure S9 | Distribution of slip planes under current stress.
Figure S10 | Void area development across various regions.
Figure S11 | Wafer packaging and preparation process.
Figure S12 | EELS Analysis of Cross-Sectional Composition.
Figure S13 | Preparation of the *in-situ* TEM sample.

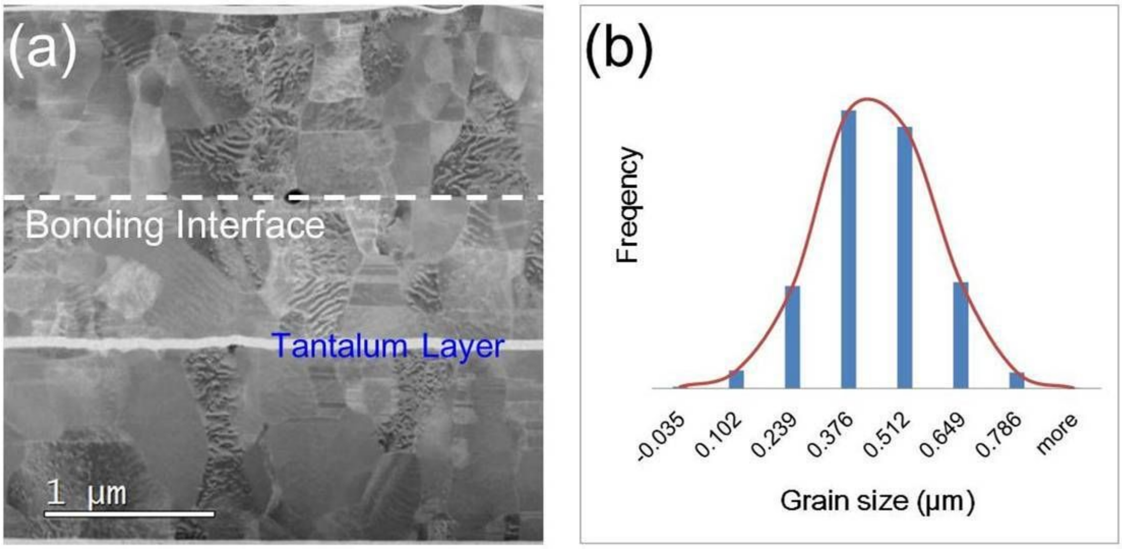

Figure S1. Average grain size of the Cu-Cu joint.

(a) STEM image of the specimen. (b) Normal distribution of grain size.

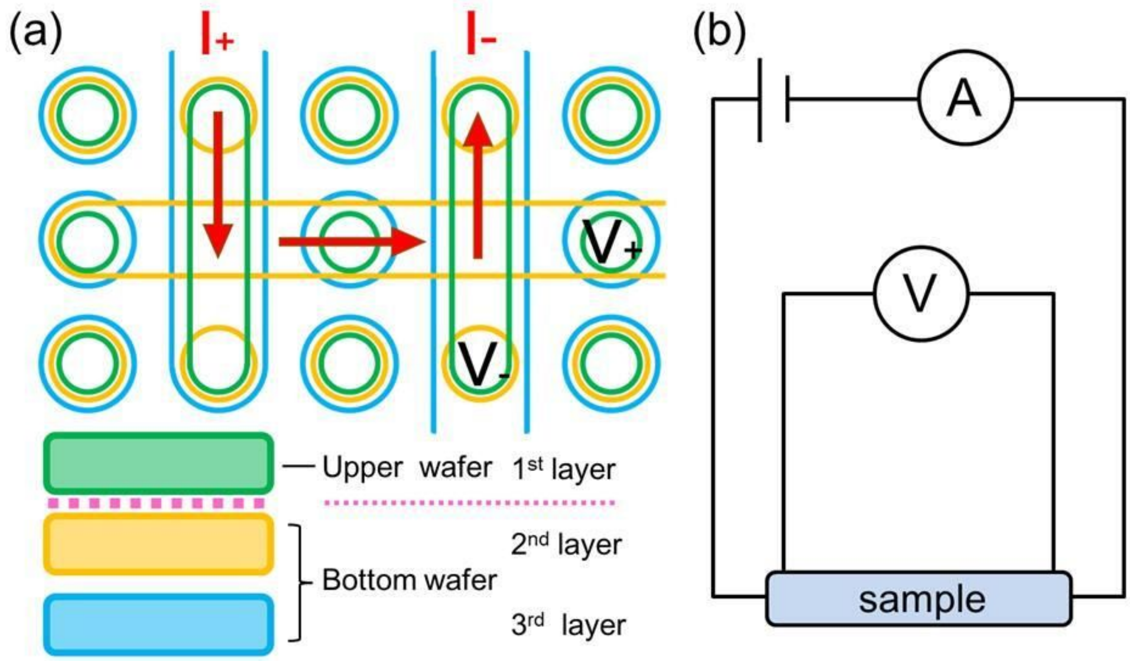

Figure S2. Kelvin structure design layout.

(a) Circuit setup of single Kelvin joint. (b) Four-point probe.

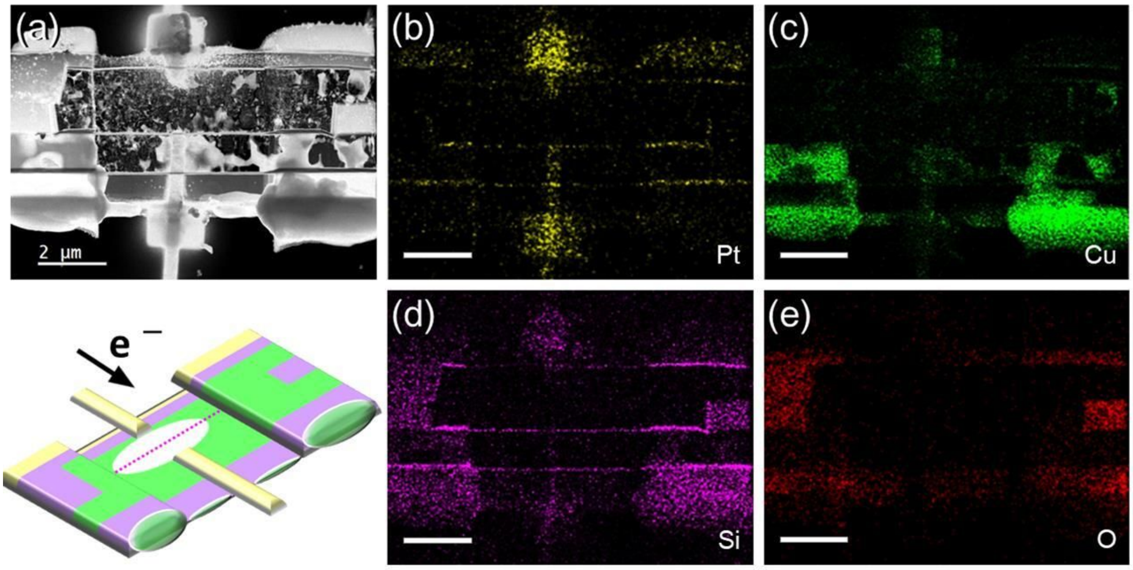

Figure S3. STEM and EDS analysis images of hillocks.

(a) STEM image of the sample after prolonged current stress. Illustration below indicates the direction of electron flow. (b–e) Element distribution on the sample.

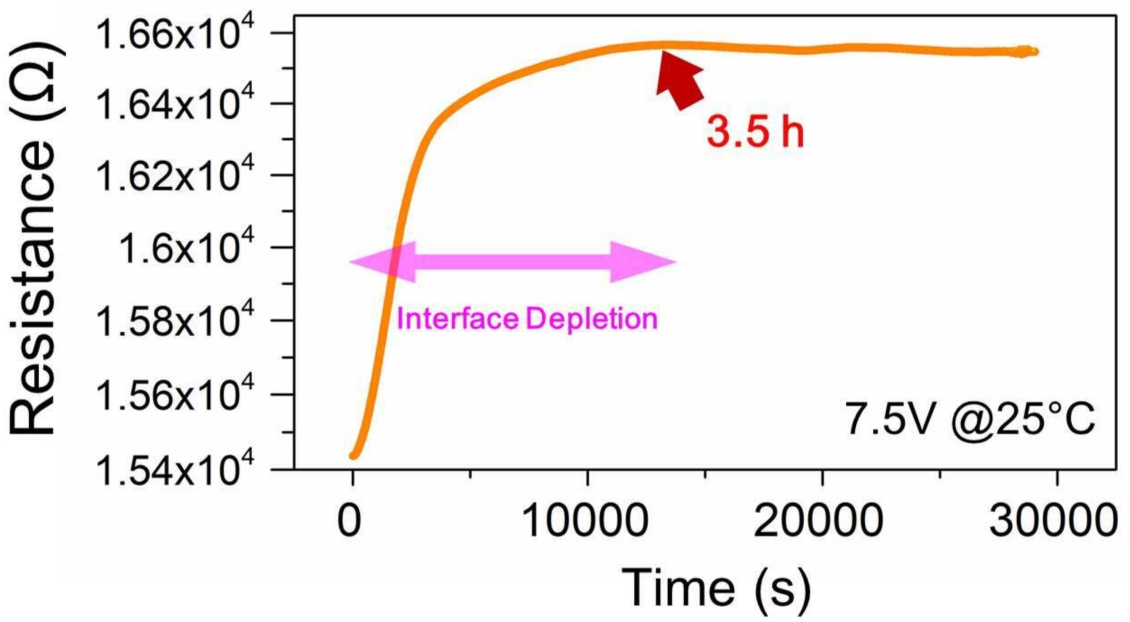

Figure S4. *Ex-situ* electrical measurement of TEM sample.

Exhibits a similar trend to the *in-situ* conditions, with a resistance growth ratio of approximately 7%. Calculated sample temperature at that time was approximately 46 °C.

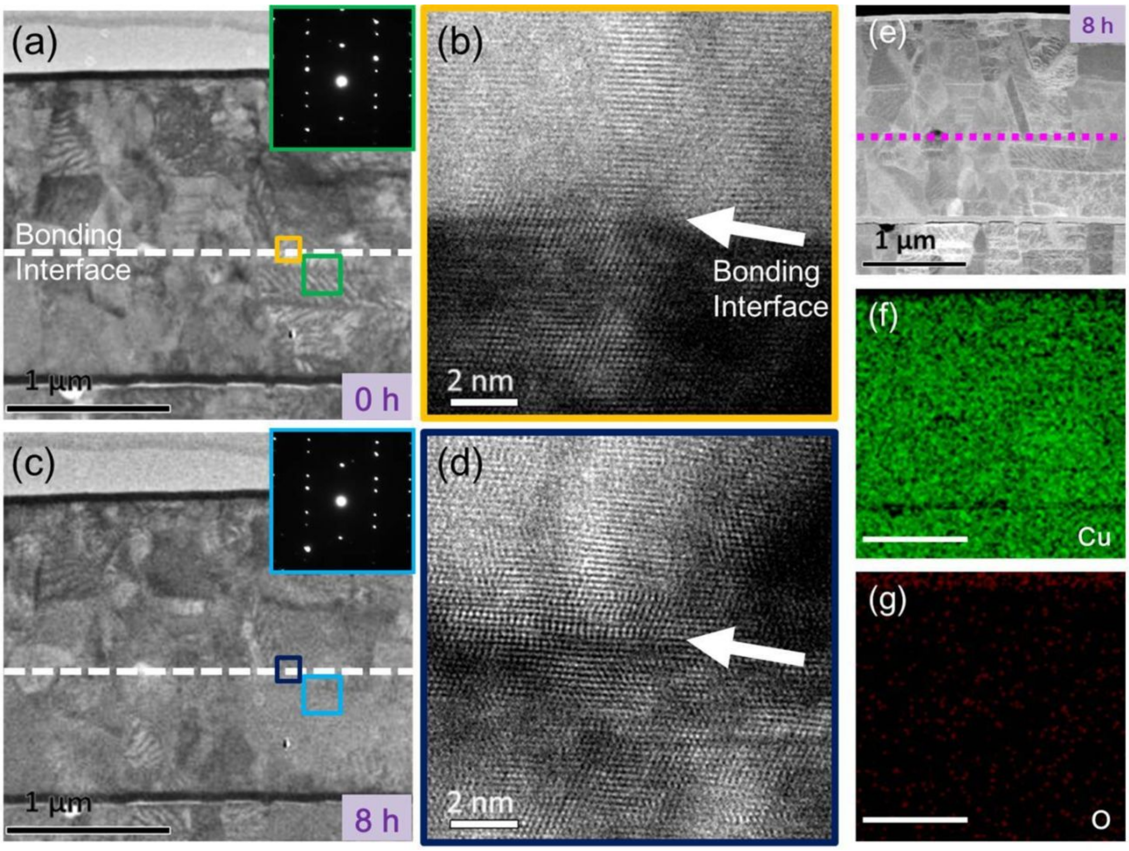

Figure S5. Characterization after prolonged holding at mild temperature.

(a–d) Bright-field TEM images of the sample at the initial state (a, b) and after 8-hour holding at 50 °C (c, d). Insets in (a) and (c) show selected area electron diffraction (SAED) patterns. HRTEM images in (b) and (d) were acquired from the marked areas in (a) and (c), respectively. (e–g) STEM-EDS analysis conducted after heating experiment.

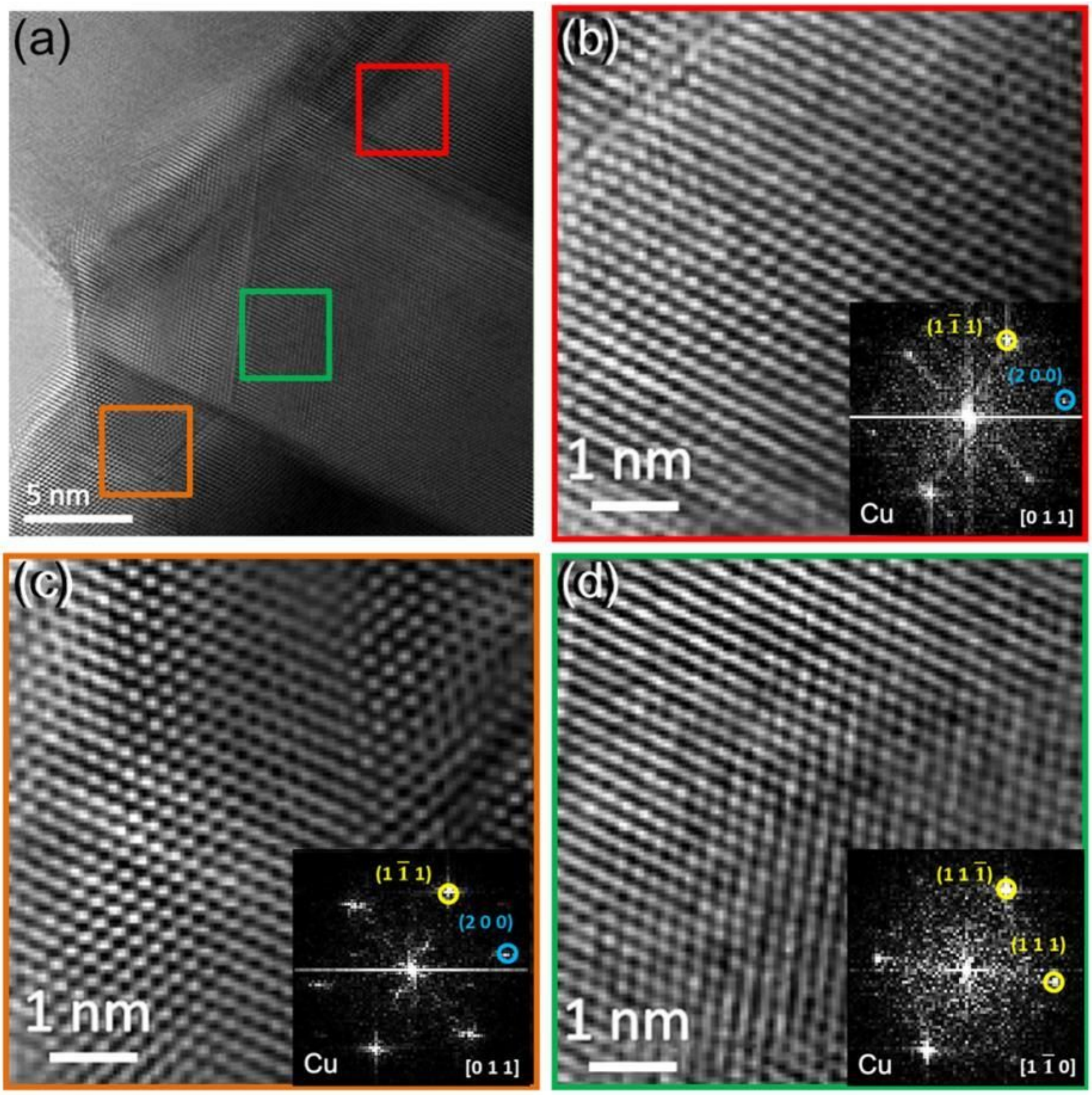

Figure S6. Atomic-scale imaging of different grains.

(a) Bright-field TEM image from Figure 3(a). (b–d) Lattice image of the area marked in (a), respectively. Each inset shows the FFT-DP of each image.

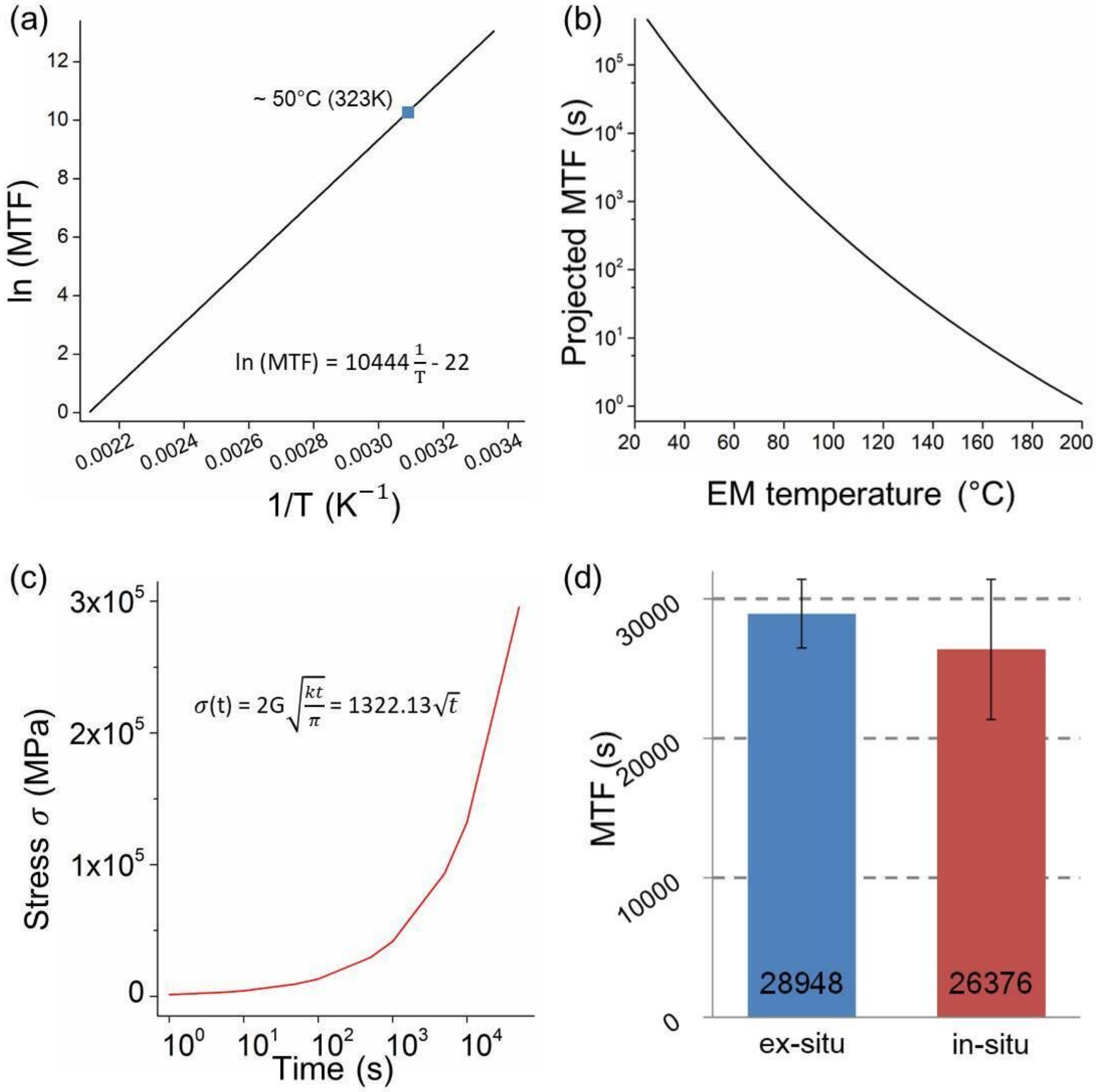

Figure S7. Model assessment and data reliability.

(a) Plot of logarithmic MTF versus reciprocal EM temperature. (b) Predicted MTF of Cu-Cu joints under EM at 10^5 A/ cm^2. (c) Stress evolution via Korhonen's model. (d) Failure time statistics of Cu-Cu joints from *in-situ* and *ex-situ* experiments.

Calculation of MTF using Black's equation

Since the *in-situ* setup was operated under a constant current and the temperature inside the vacuum chamber remained stable, Black's equation was applied to the *ex-situ* data to estimate the temperature dependence of the MTF.

Plots in (a) and (b) were derived based on the following form of Black's equation:

$$\frac{1}{\mathrm{MTF}} = \mathrm{AJ}^2 \exp - \frac{\varphi}{\mathrm{kT}}$$

which, after rearrangement, becomes:

$$\ln \mathrm{MTF} = \ln \frac{\mathrm{A}}{J^2} + \frac{\varphi}{k} \cdot \frac{1}{T}$$

The temperature T was set at 25 °C, and the MTF was obtained from the average *ex-situ* data. The actual temperature rise (~25 °C) was estimated based on the temperature coefficient of resistance (TCR).

The calculation used an activation energy φ of 0.9 eV (from previous studies), the Boltzmann constant *k* of 8.617×10^{-5} eV/ K, and a current density *J* of 10^5 A/ cm^2.

Using these values, the prefactor A was extracted and used to construct the linear plot of $\ln \mathrm{MTF}$ versus $\frac{1}{T}$, shown in (a), which illustrates the temperature dependence

of MTF under constant current conditions. Plot (b) presents the same data in a more conventional form, with MTF plotted directly against temperature (in °C).

Note: A 10% resistance increase was adopted in this calculation to enable a simplified estimation of MTF. While this choice may affect absolute values, it preserves the reliability of the temperature-dependent trend.

Stress estimation using Korhonen's model

To conceptually describe stress development in the transient regime, the Korhonen's model was considered as a theoretical reference, although its applicability to the present system is limited. Given the geometric constraints of the TEM samples, we apply the semi-infinite line solution of the model, which is valid during the early stage of current application, before the atomic flux front reaches the opposite terminal. This condition satisfies $L > \sqrt{Kt}$, where L is the interconnect length, K is the diffusivity-related constant, and t is time.

Semi-infinite line solution of the Korhonen's model:

$$\sigma(t) = 2G\sqrt{\frac{Kt}{\pi}}$$

Here, $G = \frac{q^* E}{\Omega}$ represents the driving pressure gradient for EM, where $q^* = |z^*| \cdot e$ is the effective charge, E is the electric field, and Ω is the atomic volume.

The effective diffusivity is given by $K = \frac{D_a B \Omega}{kT}$, where D_a is the diffusion coefficient at a given temperature, B is the bulk modulus, k is the Boltzmann constant, and T is the absolute temperature.

The value of z^* was adopted from literature and taken as -4. The elementary charge e was set as 1.6×10^{-19} C, and the Ω_{Cu} was 1.18×10^{-29} m^3. The electric field E was calculated from the current density using $J = \sigma_{Cu} \cdot E$, where $J = 10^5$ A/cm^2 $=$ 10^9 A/m^2, and $\sigma_{Cu} = 5.8 \times 10^7$ S/ m.

The diffusion coefficient D_a was assumed to be 1×10^{-20} m^2/ s at room temperature. The Young's modulus E_{Cu} was 110 GPa, and the bulk modulus was approximated as $B = 0.5 \cdot E_{Cu}$. The Boltzmann constant k was taken as 1.38×10^{-23} J/ K, and the temperature was 298 K. The value of π was approximated as 3.1416.

Using the above parameters and the known time dependence $\sigma(t) \propto \sqrt{t}$, the corresponding coefficient was computed.

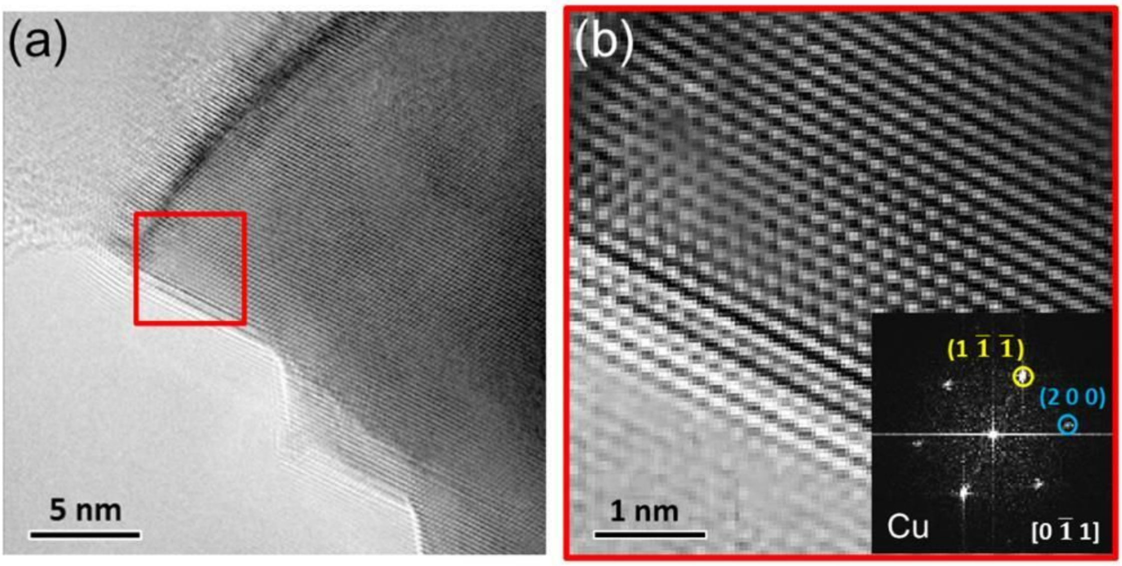

Figure S8. Crystal orientation in observation area.

(a) Bright-field TEM image from Figure 4(a). (b) HRTEM image at the location marked in (a). Inset shows the FFT-DP of (b).

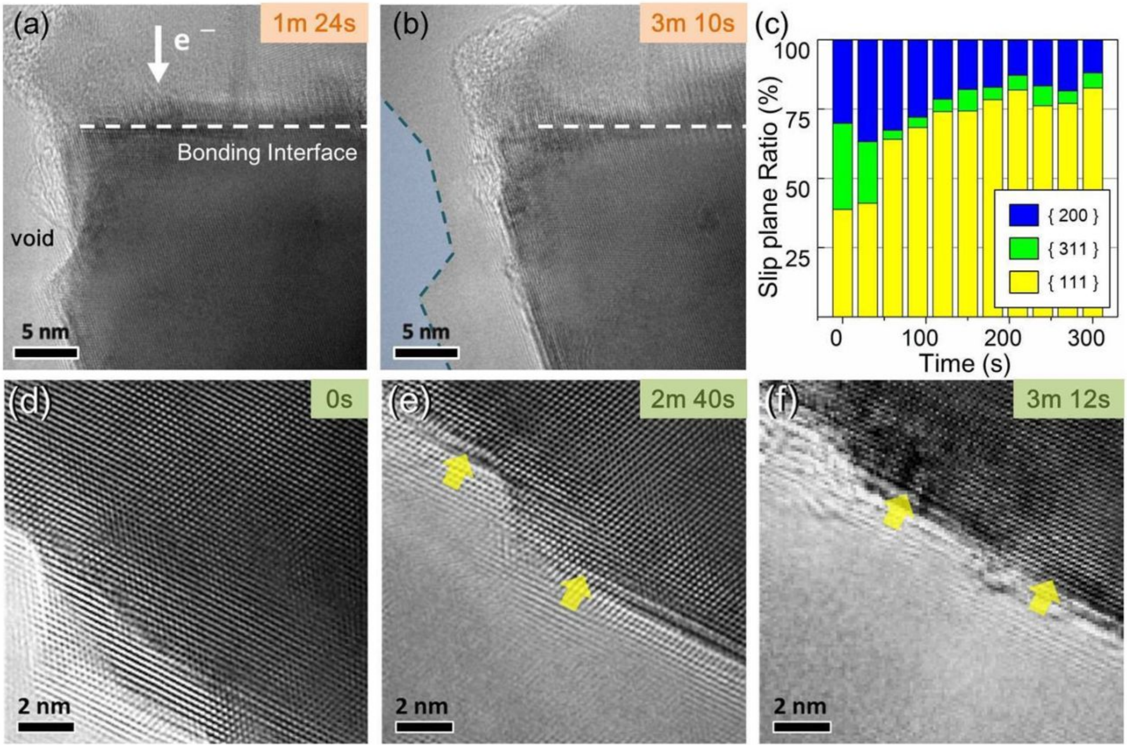

Figure S9. Distribution of slip planes under current stress.

This trend clearly demonstrates that the {111} orientation is consistently dominant.

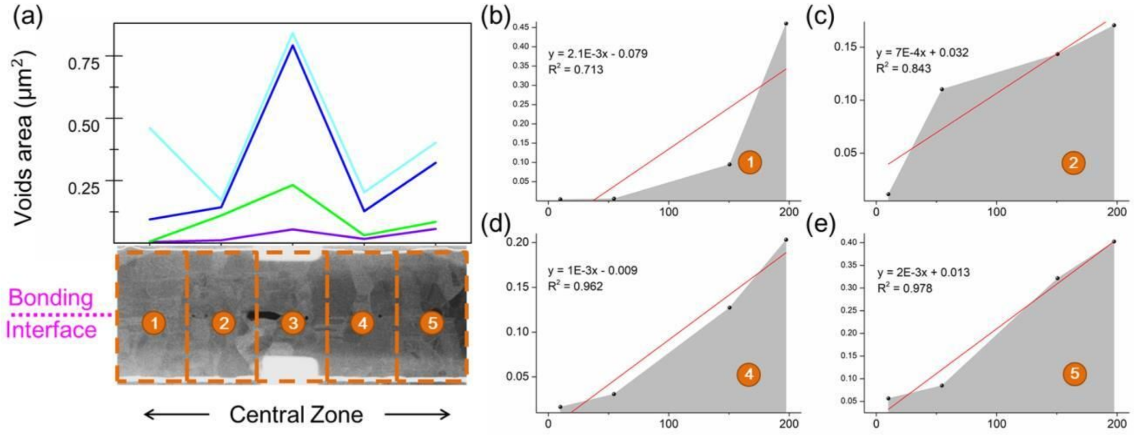

Figure S10. Void area development across various regions.

(a) Corresponding initial STEM image of Figure 5(b). (b–e) Cumulative void Growth area in different zones.

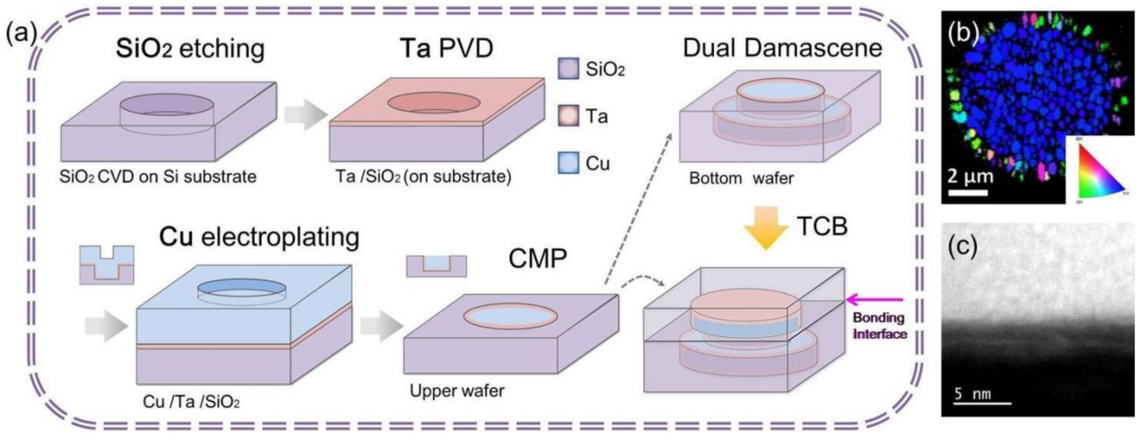

Figure S11. Wafer packaging and preparation process.

(a) Damascus inlay fabrication process. (b) EBSD mapping image of the top wafer. (c) High-resolution STEM image of the bonding interface.

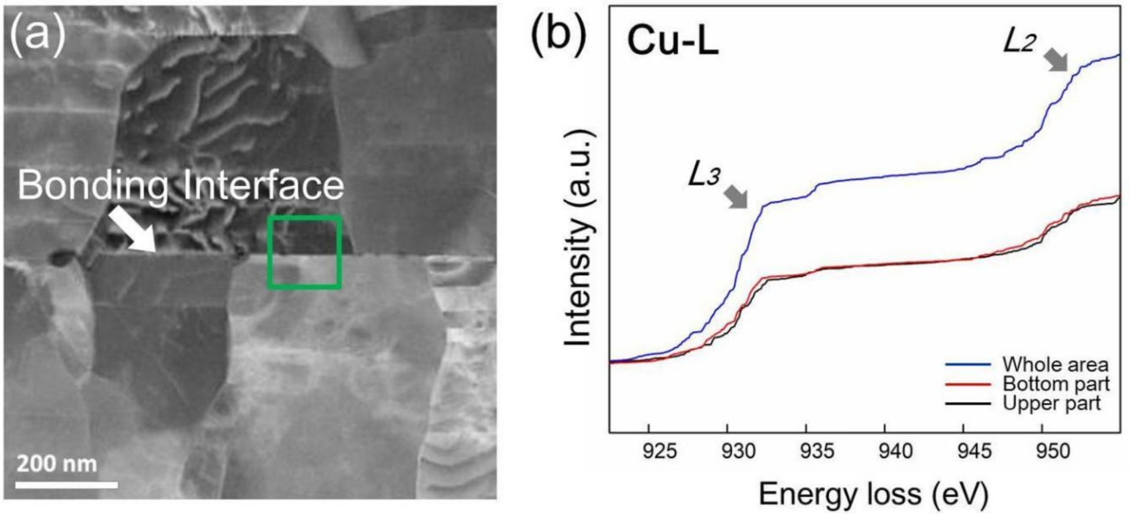

Figure S12. EELS Analysis of Cross-Sectional Composition.

(a) EELS image and selected zone of the collected signal. (b) EELS spectrum of the Cu L-edge.

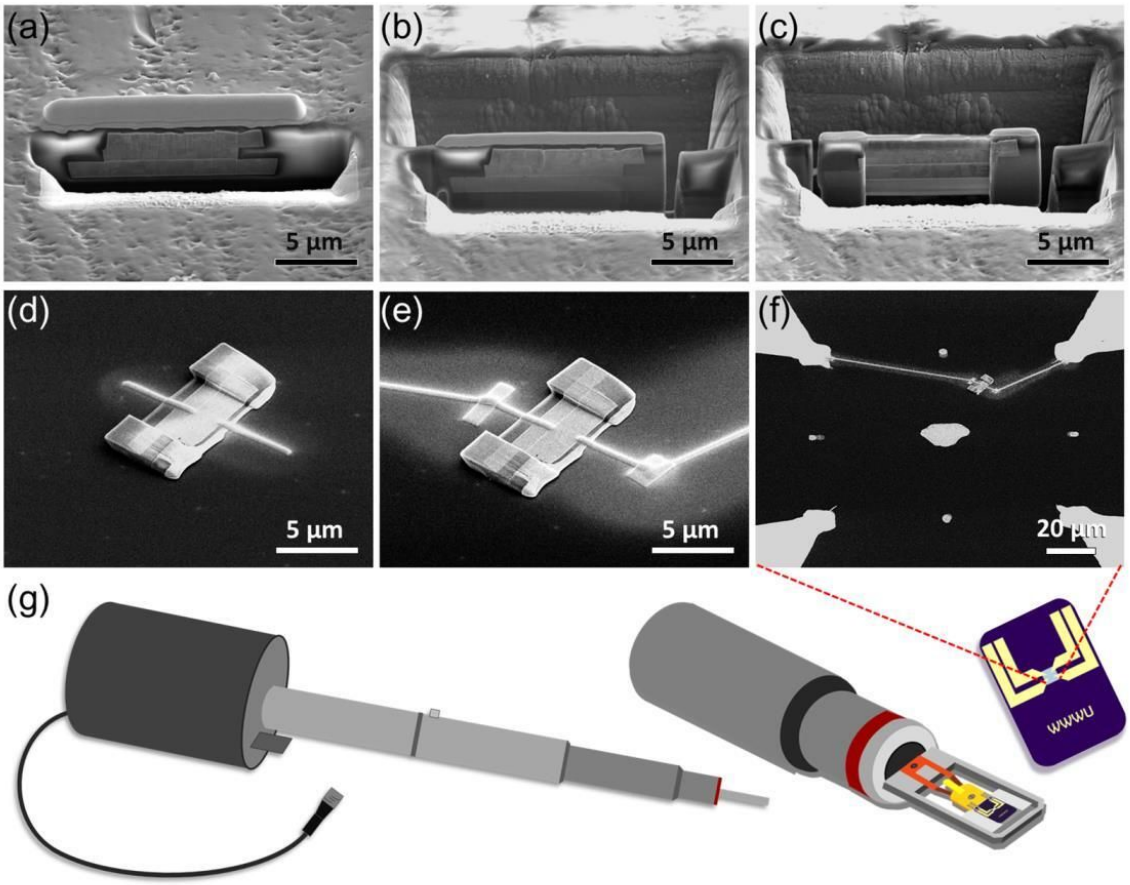

Figure S13. Preparation of the *in-situ* TEM sample.

(a) Trenching followed by the deposition of a Pt protective layer. (b) Refinement of the target area. (c) TEM lamella produced by Ga ion beam thinning. (d–f) Pt electrode deposition on the electrical chip. (g) Schematic of the electrifying *in-situ* chip and holder.
